# Peripheral inflammation is accompanied by cerebral hypoperfusion in mice

**DOI:** 10.3389/fpain.2025.1492773

**Published:** 2025-04-08

**Authors:** Afolashade Kazeem, Chuang Ge, Maral Tajerian

**Affiliations:** ^1^ Department of Biology, Queens College, City University of New York, New York City, NY, United States; ^2^ The Graduate Center, City University of New York, New York City, NY, United States

**Keywords:** complete Freud's adjuvant, laser speckle contrast imaging, animal model of pain, cerebral blood flow (CBF), inflammation

## Abstract

**Introduction:**

Chronic pain is a disabling condition that is accompanied by neuropsychiatric comorbidities such as anxiety, depression, and cognitive decline. While the peripheral alterations are well-studied, we lack an understanding of how these peripheral changes can result in long-lasting brain alterations and the ensuing behavioral phenotypes. This study aims to quantify changes in cerebral blood perfusion using laser speckle contrast imaging (LSCI) in the murine Complete Freund's adjuvant (CFA) model of unilateral peripheral inflammation.

**Methods:**

Twenty four adult male and female C57BL/6 mice were randomly assigned to control (0.05 ml saline) or 1 of 3 experimental groups receiving CFA (0.01 ml, 0.05 ml, and 0.1 ml) on the right hindpaw. Three days after the intraplantar injections, animals were examined for signs of inflammation and subjected to craniotomy and *in vivo* LSCI of the parietal-temporal lobes.

**Results:**

Unilateral administration of CFA resulted in signs of local inflammation as well as cerebral hypoperfusion in dose-dependent manner.

**Discussion:**

To our knowledge, this is the first study using laser speckle contrast imaging to examine the effects of CFA-induced peripheral inflammation on cerebral blood perfusion. It serves as a first step in delineating the path by which insult to peripheral tissues can cause long-lasting brain plasticity via vascular mechanisms.

## Introduction

Inflammation, an adaptive defensive response under homeostatic conditions, plays a crucial role in driving tissue repair and fighting likely pathogens. Pain is one of the cardinal signs of inflammation potentially caused by released mediators forming an “inflammatory soup” capable of nociceptor sensitization (1). Under pathological conditions, inflammation could lead to chronic pain often presenting with multiple co-morbid psychiatric disorders, including mood alterations (2) and cognitive impairment (3). One pivotal mechanism that could explain the chronification of pain as well as its resistance to classical treatment is the concept of pain centralization, where initial sensory events can gradually alter the central nervous system, resulting in amplified pain and/or aberrant pain that exists without peripheral sensitization. Alterations in brain circuitry have been extensively reported across a spectrum of pain conditions, such as complex regional pain syndrome (4–8), fibromyalgia (9, 10), neuropathic pain (11–17), and migraine (18), thus prompting the quest for treatments that could reset these systems. However, most of the research in this area overlooks the evolving brain microvascular changes that may parallel cellular plasticity.

The expensive and somewhat primitive nature of minimally-invasive techniques that could be used to study the pain brain in animal models have historically posed significant practical limitations. The rapid technological advances in the field of *in vivo* blood perfusion imaging paired with the high clinical relevance of such studies have resulted in renewed interest in the role of brain vascular changes and its role in linking peripheral insults to central nervous system (CNS) alterations (19), with laser speckle imaging gaining popularity as a method of *in vivo* perfusion quantitation in the clinic (20, 21). Of particular interest are cortical regions such as the somatosensory and motor cortices, both due to logistical considerations (ease of imaging of superficial structures), as well as the documented involvement of these areas in the experience of pain (22).

This study aims to quantify changes in mechanical sensitivity, cognitive function, anxiety, as well as cerebral blood perfusion using laser speckle contrast imaging (LSCI) in the murine Complete Freund's adjuvant (CFA) model of unilateral peripheral inflammation. This effort is a first step in addressing the identity of the “black box” between central and peripheral mechanism of pain, thereby opening the door to entirely novel therapeutic venues that do not only target the injured tissues but rather address the node of pain chronification.

## Materials and methods

### Animals

Twenty female and 24 male C57BL/6, 12–16 weeks of age, were purchased from a commercial supplier (Jackson lab, USA) and habituated for 14 days at the Queens College animal facility before the start of the experiment. Animals were housed in groups of 3/cage on a 12-h light/dark cycle and an ambient temperature of 20°C to 22°C, with food and water available *ad libitum*. All animal procedures were approved by the Queens College Institutional Animal Care and Use Committee (Flushing, NY, USA) and conform to the NIH guidelines (23) and the “animal subjects” guidelines of the International Association for the Study of Pain.

### Induction of peripheral inflammation

Two cohorts of mice were used: one for the behavioral experiments and the other for the imaging studies. Animals were randomly assigned to control or one of three experimental groups as described below. Normal saline or undiluted CFA (Sigma-Aldrich, Munich, Germany) were administered to the intraplantar surface of the right hindpaw under isoflurane anesthesia. Signs of hindpaw swelling and abnormal gait were noted by the observer as qualitative measures 3 days after the unilateral injections. Certain injury models may present challenges to blinding due to the potential for group identity indicators, particularly when the affected limb is directly examined, as seen in the von Frey assay. In the behavioral studies, data from the Y-maze, zero maze, and open field tests were collected via video recording and subsequently analyzed. Given that the recordings were captured from a top-down perspective, the risk of inadvertently observing paw swelling or other confounding factors was minimized. For the imaging studies, animals were assigned random identifiers, and the image analysis was conducted blindly using automated software.

(a) Control group: 0.05 ml of normal saline; *n* = 11. (b) Experimental groups: Group 1: 0.01 ml CFA; *n* = 11. Group 2: 0.05 ml CFA; *n* = 11. Group 3: 0.1 ml CFA; *n* = 11.

### Behavioral testing

All testing was carried out 3 days after treatment. All analyses were blinded to the identity and experimental condition of the animal. Mice were habituated to the testing room for 1hr prior to the start of the experiments. All behavioral apparatuses were cleaned and deodorized using 0.325% acetic acid (v/v). All videos were recorded using GoPro cameras and analyzed using the automatic animal tracking software Behaviorcloud©.

The following groups were used: (a.) Control group: 0.05 ml of normal saline; *n* = 5. (b.) Experimental groups: Group 1: 0.01 ml CFA; *n* = 5. Group 2: 0.05 ml CFA; *n* = 5. Group 3: 0.1 ml CFA; *n* = 5.

#### Mechanical sensitivity

Calibrated monofilaments (Stoelting Co., USA) were applied to the plantar surface of the hind paw and the 50% threshold to withdraw (grams) was calculated as previously described (24). The stimulus intensity ranged from 0.004 to 1.7 g, corresponding to filament numbers 1.65, 2.36, 2.44, 2.83, 3.22, 3.61, 3.84, 4.08, 4.17, and 4.31. For each animal, the actual filaments used within the aforementioned series were determined based on the lowest filament to evoke a positive response (response = flexion reflex) followed by five consecutive stimulations using the up–down method. The filament range and average interval were then incorporated along with the response pattern into each individual threshold calculation.

#### Y-maze

Rodents' natural inclination to explore new environments was evaluated by quantifying spontaneous alternation in exploring the arms of the maze. In a Y-shaped maze with three identical arms (A, B, and C), mice tend to choose a new arm over one they've already visited. The maze, was made in-house using opaque white acrylic [arm dimensions: 37.4 cm × 7.6 cm × 23.3 cm (LXWXH)]. Mice were placed in the center and allowed to explore for 5 min. Mice with strong spatial working memory typically entered a new arm without revisiting a previous one. The test measured spontaneous alternation by tracking unique sequences of arm entries (e.g., ABC, BCA). The percentage of alternation was calculated by dividing the number of unique sequences by the total arm entries minus two.

#### Zero maze

The apparatus was made in-house and had the following dimensions: inner circle diameter = 47 cm, outer circle diameter = 54 cm, height = 25 cm. Mice were placed at the boundary between the open and enclosed regions and allowed to explore freely for six minutes. The time spent by the mice in each of these regions was quantified.

#### Open field

The apparatus was built in-house as a 28 cm × 28 cm × 28 cm (LXWXH) acrylic cube (light intensity = 130.2 lux at arena level). Mice were placed in the center of the field and allowed to explore for 6 min. The time spent in the central 10% and 25% areas vs. the entire arena was calculated in addition to the speed of locomotion in the central and peripheral areas.

### Craniotomy

Mice were weighed and dexamethasone (5 ml/mg, i.p.) was administered to prevent/reduce the occurrence of cerebral edema caused by the side effects of isoflurane and the impact of drilling the skull. Under isoflurane anesthesia, mice were transferred to a stereotaxic frame and positioned on a heating pad with continuous temperature monitoring, with sterile ointment applied to the eyes to prevent dryness. The scalp was shaved and cleaned with povidone before incision and drilling (drill bit diameter = 0.8 mm). The mouse was monitored continuously during surgery, the drill bit was cooled intermittently, and the bone and tissues were moistened with normal saline to prevent dryness. A skull window was created, exposing the cerebrum's left and right parietal-temporal lobes.

### Laser speckle contrast imaging

The following groups were used: (a.) Control group: 0.05 ml of normal saline; *n* = 6. (b.) Experimental groups: Group 1: 0.01 ml CFA; *n* = 6. Group 2: 0.05 ml CFA; *n* = 6. Group 3: 0.1 ml CFA; *n* = 6.

The Laser speckle imaging system was used to capture and analyze blood flow dynamics (17) in the brain tissue 3 days post-CFA/saline administration. The setup includes a coherent laser light source, a camera for capturing speckle patterns, and RFLSI iii computer software for data analysis (RWD Life Sciences, Shenzhen, China). Following the skull window establishment, the mouse brain tissue was exposed to laser intensity of 80 mw, and white light intensity was level 4 for a recording duration of 12 s. The exposure time was 8 ms, and the HD temporal algorithm was 2048*2048. Blood perfusion data was analyzed for a constant regions across all animals (0.7cm × 0.5 cm) using the RFLSI iii software.

### Statistical analysis

Analysis of behavioral data was carried out using either one-way analysis of variance (ANOVA, y-maze, zero-maze, open field, cerebral perfusion) or two-way repeated measures ANOVA (von Frey) followed by Bonferroni's multiple comparison test. The Brown–Forsythe test was used to assess equality of variances among groups (standard deviations were not significantly different among the groups for any of the datasets). Routs outlier test (*Q* = 1.0%) was used to identify statistical outliers (no outliers were detected). Significance was set at *P* value <0.05; GraphPad Prism V8.0 (GraphPad Software, San Diego, USA).

## Results

Unilateral injection of CFA into the mouse hindpaw resulted in classic signs of inflammation per the subjective observation of the experimenter 3 days after treatment. The administration of saline was not linked to signs of hindpaw swelling or abnormal gait. Meanwhile, the low dose of 0.01 ml CFA caused mild swelling in the right paw and abnormal gait, the medium dose of 0.05 ml CFA resulted in swelling extending beyond the paw as well as abnormal gait, and the high dose of 0.1 ml caused swelling extending farther beyond the paw and abnormal gait.

**Figure 1:**
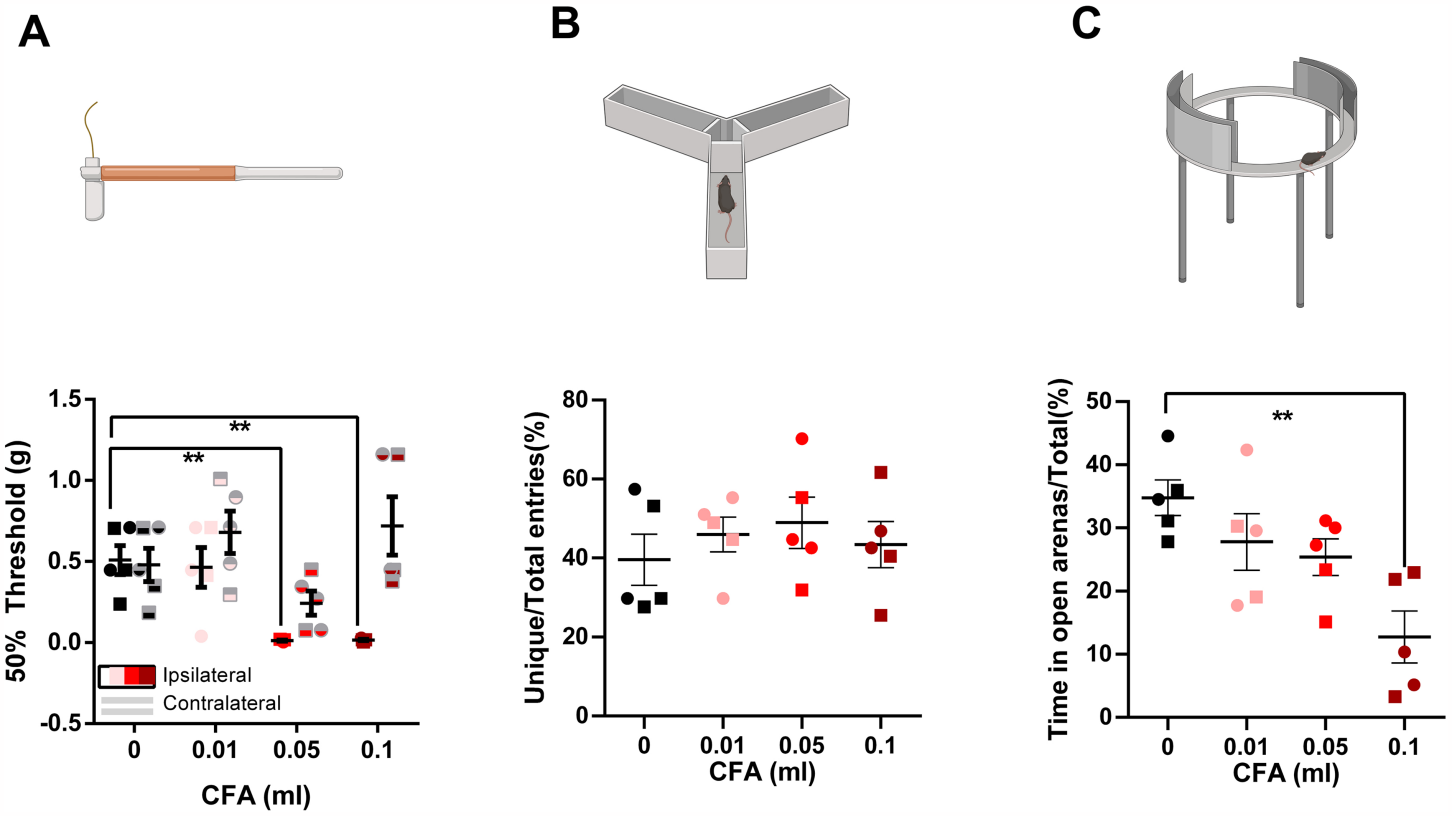
Visual illustration of the study paradigms demonstrating the control (saline) and the 3 experimental groups in the von frey, y-maze, and zero-maze assays. **(A)** Compared to the saline-treated group, animals receiving 0.05 ml or 0.1 ml of CFA demonstrated reduced mechanical sensitivity thresholds on the affected hindpaw. No differences between groups were observed for the contralateral measurements (two-way repeated-measures ANOVA followed by Bonferroni's test for multiple comparisons). **(B)** No differences were observed in the % of unique entries in the y-maze. **(C)** Animals receiving 0.1 ml of CFA spent less time in the open arenas of the zero-maze (one-way ANOVA followed by Bonferroni's test for multiple comparisons). ○ indicates females and □ indicates males. *n* = 5/group; error bars indicate S.E.M. Figure created using BioRender©.

To provide a less subjective assessment of behavioral changes related to paw inflammation, we conducted tests measuring mechanical sensitivity (von Frey), working memory (y-maze), anxiety-like behaviors (zero maze, open field), and overall locomotor function (open field). Our results indicate a significant increase in mechanical sensitivity following CFA administration where, both groups receiving 0.05 ml or 0.1 ml of CFA demonstrated reduced mechanical thresholds on the ipsilateral paw when compared to the control group. No differences between groups were observed for the contralateral measurements [Figure 1A; CFA dose × Paw laterality factors interaction % of total variation = 16, *P* = 0.02, *F* (3, 16) = 4.358; CFA dose % of total variation  = 25.74, *p* = 0.004, *F* (3, 16) = 6.590; Paw laterality factor % of total variation = 17.85, *p* = 0.0015, *F* (1, 16) = 14.58; 0 ml CFA ipsilateral vs. 0.05 ml CFA ipsilateral mean difference = 0.496, adjusted *p* value = 0.007, 95% CI of diff. = 0.1199–0.8723; 0 ml CFA ipsilateral vs. 0.1 ml CFA ipsilateral mean difference = 0.492, adjusted *p* value = 0.007, 95% CI of diff. = 0.1158–0.8682; two-way repeated measures ANOVA followed by Bonferroni's test for multiple comparisons].

**Figure 2:**
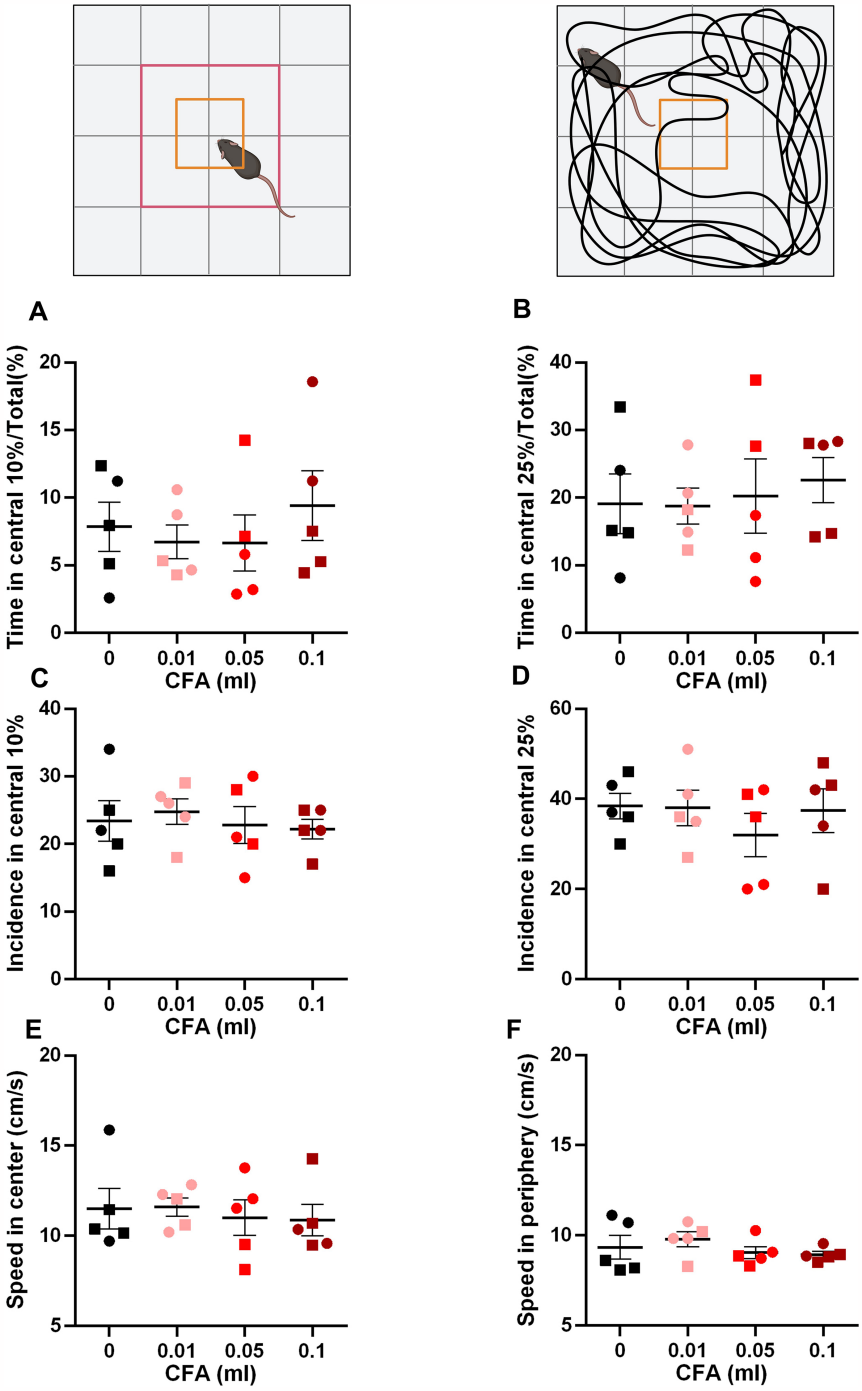
Visual illustration of the study paradigm demonstrating the apparatus and the parameters measured. **(A,B)** No differences were observed in the time spent in the central 10% **(A)** or 25% **(B)** arena. **(C,D)** No differences were observed in the incidence of entry to the central 10% **(C)** or 25% **(D)** arena. **(E,F)** No differences were observed in the speed of locomotion in the central **(E)** or peripheral **(F)** arenas of the apparatus (one-way ANOVA). ○ indicates females and □ indicates males. *n* = 5/group; error bars indicate S.E.M. Figure created using BioRender©.

We did not detect any deficits in working memory using the y-maze test [Figure 1B, one-way ANOVA *p* = 0.714, *F* (3, 16) = 0.4597]. However, we detected signs of anxiety-like behaviors in the 0.1 ml CFA group compared to control in the zero maze assay [Figure 1C, one-way ANOVA *p* = 0.005, *F* (3, 16) = 6.376; Bonferroni's multiple comparison test with an adjusted *P* value of 0.002, mean difference = 22.07, 95.00% CI of diff. = 8.282–35.87]. Surprisingly, anxiety-like behavior was not detected in the open field assay, as measured by % time spend in the central 10% area [Figure 2A, one-way ANOVA *p* = 0.870, *F* (3, 16) = 0.2361] or central 25% area [Figure 2B, one-way ANOVA *p* = 0.909, F (3, 16) = 0.1790]. Similarly, we did not detect differences in the incidence of central area entry [Figure 2C, one-way ANOVA *p* = 0.88, *F* (3, 16) = 0.222] for central 10% area and Figure 2D, one-way ANOVA *p* = 0.68, *F* (3, 16) = 0.52. It is noteworthy that due to the small size of the open field apparatus, it is not one that is anxiogenic in control animals.

Since many behavioral assays rely on motor function, we measured the speed of locomotion in the central and peripheral areas of the open field. No differences were observed in any of the groups (Figures 2E,F). Central area: one-way ANOVA *p* = 0.92, *F* (3, 16) = 0.159 and peripheral area: one-way ANOVA *p* = 0.519, *F* (3, 16) = 0.785.

**Figure 3:**
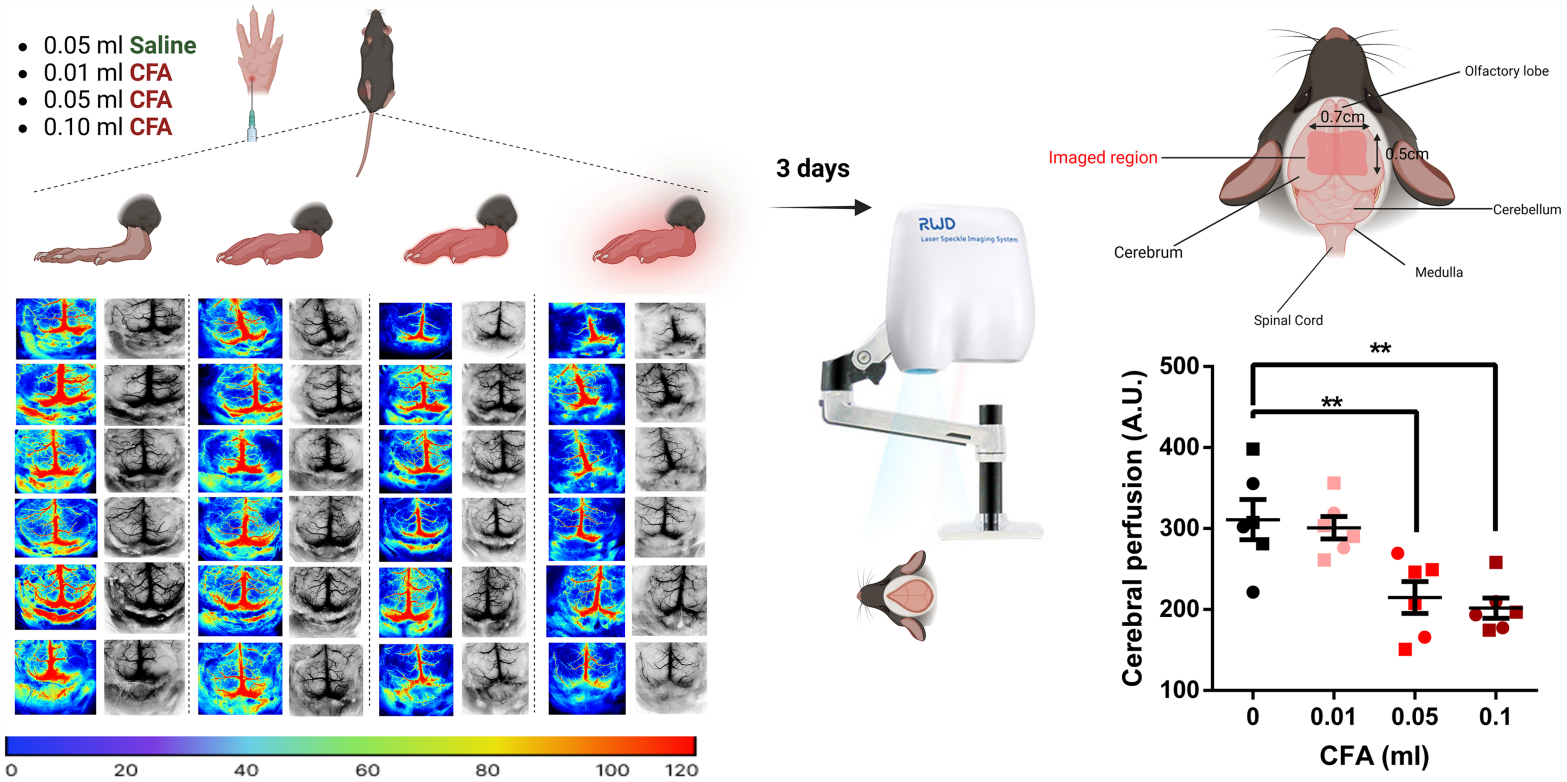
Visual illustration of the study paradigm demonstrating the control (saline) and the 3 experimental groups with the respective P-color images and gray cerebral blood flow patterns in twenty-four mice brains 3 days post-CFA/saline injection. Groups receiving 0.05 ml or 0.1 ml of CFA demonstrated reduced levels of cerebral perfusion (one-way ANOVA followed by Bonferroni's test for multiple comparisons). ○ indicates females and □ indicates males. *n* = 6/group; error bars indicate S.E.M. Figure created using BioRender©.

Peripheral inflammation was associated with cerebral hypoperfusion in the parietal-temporal lobes in a dose-dependent manner 3 days after CFA administration [Figure 3, one-way ANOVA, *p* = 0.0004, *F* (3, 20) = 9.52]. Compared to the saline control, 0.05 ml and 0.1 ml CFA-treated groups demonstrated reduced perfusion rates (Bonferroni's multiple comparison test with an adjusted *P* value of 0.004, mean difference = 96.19, 95% CI of diff. = 28.17–164.2 for the 0.05 ml group and an adjusted *P* value of 0.001, mean difference = 109.3, 95% CI of diff. = 41.27–177.3 for the 0.1 ml group).

## Discussion

The CFA model of inflammation, while limited in its duration and severity of symptoms, is valuable in demonstrating the link between peripheral mechanisms of painful injury and the ensuing behavioral changes and biochemical and molecular alterations in central nervous system tissues. For example, hindpaw inflammation (0.02 ml CFA) has been associated with stimulus-evoked hypersensitivity as well as measures of voluntary behavior alterations up to 9 days post-injection (25). Additionally, CFA (0.05 ml) administration in the rat hindpaw resulted in increased anxiety, increased levels of circulating corticosterone as well as decreased global DNA methylation levels in the amygdala 10 days after treatment (26). In a separate study using in the same model, signs of increased blood-brain-barrier (BBB) permeability were paralleled by alterations in transmembrane tight junction protein levels (27). Our results complement these observations by showing CFA-associated pain and anxiety as well as dose-dependent cerebral hypoperfusion 3 days following the onset of inflammation.

These findings can be viewed within the wider scope of peripheral inflammation using different models. For example, the murine lipopolysaccharide (LPS) model has repeatedly been associated with cognitive dysfunction (28) as well as aberrant synaptic phagocytosis by microglia (29), even prompting the hypothesis that LPS exposure could be a contributing factor to neurodegenerative disorders such as Alzheimer's disease (30). Inflammation subsequent to systemic LPS has also shown to be accompanied by ultrastructural cyto-architechtural changes in the BBB (31). Finally, reduced perivascular flow of cerebrospinal fluid, in the absence of changes in blood flow, was observed in a murine LPS model (32). There is some evidence for clinical translation as well: in patients with chronic neck and upper body pain, neck disability indices predicted cerebral hypoperfusion measured by single-photon emission computed tomography, with higher indices being linked to greater hypoperfusion (33). In the more extreme case of sepsis, behavioral changes such as delirium and cognitive decline are linked to changes in cerebral microcirculation. For instance, both perfused cerebral vessel density and perfused small vessels were decreased in a ovine model of septic shock due to peritonitis (34).

While our presented data is at the “proof of concept” stage, we plan on exploring the hypothesis that alterations in cerebral perfusion could present as a mechanistic link between peripheral inflammation and brain plasticity, and there is evidence for bidirectional modulation between the two. In models of chronic cerebral hypoperfusion, the extent of hypoperfusion is correlated to cognitive deficits (35) and vascular dementia is observed along with loss of BBB integrity, with a recent study demonstrating a role for the mechanosensitive piezo1 channel (36).

The adaptive value of cerebral hypoperfusion after peripheral inflammation remains uncertain. It is possible that hypoperfusion may aid in the minimization of inflammogen entry to the brain (37). Similar to our findings, cerebral microcirculation was shown to be impaired in an ovine model of experimental peritonitis (38). Since peripheral LPS is associated with changes in the BBB (31) the body might attempt to protect the brain by limiting the infiltration of peripheral inflammatory mediators, thereby preserving BBB integrity/limiting BBB damage and reducing the likelihood of further neuroinflammatory processes. It is also possible that it is secondary/compensatory instrument to systemic metabolic change since systemic inflammation in mice (LPS model) is associated with increased cerebral oxygen demand (39). This “Metabolic hypothesis” (40) should be considered alongside the “neurogenic hypothesis” where specific neuronal populations may guide the vascular response (41), especially since they could be exerting opposing effects (42). The current experimental paradigm does not examine neuronal activity or cellular energy demand in the context of cerebral blood flow alterations. It is possible to study these variables concurrently, for example by adopting an electrocorticography-LCSI approach as shown in models of epilepsy (43). Furthermore, future studies will target specific regions of interest, compare the right vs. left hemispheres, and study timepoints beyond the 3-day window explored in this manuscript.

The current study includes male and female subjects without directly comparing the 2 sexes. This is due to the following reasons: first, comparative data from male and female C57BL/6 mice showed no differences in anxiety-like behavior, depression-like behavior, and cognitive abilities, with equivalent between-group variances (44). This is reflected in our current work where disaggregating data by sex did not result in any significant changes. Second, our brief research report serves to give an overview of this novel imaging method applied to a model of peripheral inflammation. Future studies employing a factorial design with sex as a factor will be used. As it stands, the current study is not powered to detect sex differences. Third, the NIH mandate of considering sex as a biological variable is not the same as searching for sex differences. Indeed, the relevant NIH policies do not to require the doubling of the sample size nor do they require researchers to carry out power studies to detect sex differences. The goal is to include females in preclinical studies and show the data in a transparent manner (45).

To our knowledge, this is the first study using laser speckle contrast imaging to examine the effects of CFA-induced peripheral inflammation on cerebral blood perfusion. The findings of decreased perfusion with increasing dose of CFA are useful in providing a mechanistic link between central and peripheral tissues in the processing of pain, and can provide insight into the underpinnings of centralization/chronification in other types of painful peripheral injuries.

## Data Availability

The original contributions presented in the study are included in the article/Supplementary Material, further inquiries can be directed to the corresponding author.

## References

[1] Cook AD Christensen AD Tewari D McMahon SB Hamilton JA. Immune cytokines and their receptors in inflammatory pain. Trends Immunol. (2018) 39(3):240–55. 10.1016/j.it.2017.12.003 29338939

[2] McWilliams LA Goodwin RD Cox BJ. Depression and anxiety associated with three pain conditions: results from a nationally representative sample. Pain. (2004) 111(1–2):77–83. 10.1016/j.pain.2004.06.002 15327811

[3] Berryman C Stanton TR Jane Bowering K Tabor A McFarlane A Lorimer Moseley G. Evidence for working memory deficits in chronic pain: a systematic review and meta-analysis. Pain. (2013) 154(8):1181–96. 10.1016/j.pain.2013.03.002 23707355

[4] Geha PY Baliki MN Harden RN Bauer WR Parrish TB Apkarian AV. The brain in chronic CRPS pain: abnormal gray-white matter interactions in emotional and autonomic regions. Neuron. (2008) 60(4):570–81. 10.1016/j.neuron.2008.08.022 19038215 PMC2637446

[5] Seifert F Kiefer G DeCol R Schmelz M Maihofner C. Differential endogenous pain modulation in complex-regional pain syndrome. Brain. (2009) 132(Pt 3):788–800. 10.1093/brain/awn346 19153154

[6] Tajerian M Alvarado SG Clark JD. Differential olfactory bulb methylation and hydroxymethylation are linked to odor location memory bias in injured mice. Mol Pain. (2019) 15:1744806919873475. 10.1177/1744806919873475 31407613 PMC6712758

[7] Tajerian M Hung V Nguyen H Lee G Joubert LM Malkovskiy AV The hippocampal extracellular matrix regulates pain and memory after injury. Mol Psychiatry. (2018) 23(12):2302–13. 10.1038/s41380-018-0209-z 30254235 PMC6294737

[8] Tajerian M Leu D Zou Y Sahbaie P Li W Khan H Brain neuroplastic changes accompany anxiety and memory deficits in a model of complex regional pain syndrome. Anesthesiology. (2014) 121(4):852–65. 10.1097/ALN.0000000000000403 25093591 PMC4175292

[9] Harris RE Sundgren PC Craig AD Kirshenbaum E Sen A Napadow V Elevated insular glutamate in fibromyalgia is associated with experimental pain. Arthritis Rheum. (2009) 60(10):3146–52. 10.1002/art.24849 19790053 PMC2827610

[10] Napadow V Kim J Clauw DJ Harris RE. Decreased intrinsic brain connectivity is associated with reduced clinical pain in fibromyalgia. Arthritis Rheum. (2012) 64(7):2398–403. 10.1002/art.34412 22294427 PMC3349799

[11] Alvarado S Tajerian M Millecamps M Suderman M Stone LS Szyf M. Peripheral nerve injury is accompanied by chronic transcriptome-wide changes in the mouse prefrontal cortex. Mol Pain. (2013) 9:21. 10.1186/1744-8069-9-21 23597049 PMC3640958

[12] Alvarado S Tajerian M Suderman M Machnes Z Pierfelice S Millecamps M An epigenetic hypothesis for the genomic memory of pain. Front Cell Neurosci. (2015) 9:88. 10.3389/fncel.2015.00088 25852480 PMC4371710

[13] Becerra L Morris S Bazes S Gostic R Sherman S Gostic J Trigeminal neuropathic pain alters responses in CNS circuits to mechanical (brush) and thermal (cold and heat) stimuli. J Neurosci. (2006) 26(42):10646–57. 10.1523/JNEUROSCI.2305-06.2006 17050704 PMC6674763

[14] Cauda F D'Agata F Sacco K Duca S Cocito D Paolasso I Altered resting state attentional networks in diabetic neuropathic pain. J Neurol Neurosurg Psychiatry. (2010) 81(7):806–11. 10.1136/jnnp.2009.188631 19955113

[15] Cauda F Sacco K Duca S Cocito D D'Agata F Geminiani GC Altered resting state in diabetic neuropathic pain. PLoS One. (2009) 4(2):e4542. 10.1371/journal.pone.0004542 19229326 PMC2638013

[16] DaSilva AF Becerra L Pendse G Chizh B Tully S Borsook D. Colocalized structural and functional changes in the cortex of patients with trigeminal neuropathic pain. PLoS One. (2008) 3(10):e3396. 10.1371/journal.pone.0003396 18923647 PMC2561059

[17] Tajerian M Alvarado S Millecamps M Vachon P Crosby C Bushnell MC Peripheral nerve injury is associated with chronic, reversible changes in global DNA methylation in the mouse prefrontal cortex. PLoS One. (2013) 8(1):e55259. 10.1371/journal.pone.0055259 23383129 PMC3557255

[18] Borsook D Hargreaves R. Brain imaging in migraine research. Headache. (2010) 50(9):1523–7. 10.1111/j.1526-4610.2010.01761.x 20958298

[19] Zhang D Wang W Zhu X Li R Liu W Chen M Epinephrine-induced effects on cerebral microcirculation and oxygenation dynamics using multimodal monitoring and functional photoacoustic microscopy. Anesthesiology. (2023) 139(2):173–85. 10.1097/ALN.0000000000004592 37079748 PMC11672663

[20] Dimanche A Miller DR Goldberg J Raabe A Dunn AK Bervini D. Continuous hemodynamics monitoring during arteriovenous malformation microsurgical resection with laser speckle contrast imaging: case report. Front Surg. (2023) 10:1285758. 10.3389/fsurg.2023.1285758 38162090 PMC10757834

[21] Parthasarathy AB Weber EL Richards LM Fox DJ Dunn AK. Laser speckle contrast imaging of cerebral blood flow in humans during neurosurgery: a pilot clinical study. J Biomed Opt. (2010) 15(6):066030. 10.1117/1.3526368 21198204 PMC9113397

[22] Kitano K O'Hashi K Fujita S Kobayashi M. Reduction in calcium responses to whisker stimulation in the primary somatosensory and motor cortices of the model mouse with trigeminal neuropathic pain. J Oral Biosci. (2024) 66(3):587–93. 10.1016/j.job.2024.06.003 38880250

[23] Guide for the Care and Use of Laboratory Animals. Washington (DC) (2011).

[24] Chaplan SR Bach FW Pogrel JW Chung JM Yaksh TL. Quantitative assessment of tactile allodynia in the rat paw. J Neurosci Methods. (1994) 53(1):55–63. 10.1016/0165-0270(94)90144-9 7990513

[25] Pitzer C Kuner R Tappe-Theodor A. Voluntary and evoked behavioral correlates in inflammatory pain conditions under different social housing conditions. Pain Rep. (2016) 1(1):e564. 10.1097/PR9.0000000000000564 29392189 PMC5741310

[26] Spinieli RL Cazuza RA Sales AJ Carolino ROG Martinez D Anselmo-Franci J Persistent inflammatory pain is linked with anxiety-like behaviors, increased blood corticosterone, and reduced global DNA methylation in the rat amygdala. Mol Pain. (2022) 18:17448069221121307. 10.1177/17448069221121307 35974687 PMC9393577

[27] Brooks TA Hawkins BT Huber JD Egleton RD Davis TP. Chronic inflammatory pain leads to increased blood-brain barrier permeability and tight junction protein alterations. Am J Physiol Heart Circ Physiol. (2005) 289(2):H738–743. 10.1152/ajpheart.01288.2004 15792985 PMC4638185

[28] Shaw KN Commins S O'Mara SM. Lipopolysaccharide causes deficits in spatial learning in the watermaze but not in BDNF expression in the rat dentate gyrus. Behav Brain Res. (2001) 124(1):47–54. 10.1016/S0166-4328(01)00232-7 11423165

[29] Manabe T Racz I Schwartz S Oberle L Santarelli F Emmrich JV Systemic inflammation induced the delayed reduction of excitatory synapses in the CA3 during ageing. J Neurochem. (2021) 159(3):525–42. 10.1111/jnc.15491 34379806

[30] Brown GC Heneka MT. The endotoxin hypothesis of Alzheimer’s disease. Mol Neurodegener. (2024) 19(1):30. 10.1186/s13024-024-00722-y 38561809 PMC10983749

[31] Erickson MA Shulyatnikova T Banks WA Hayden MR. Ultrastructural remodeling of the blood-brain barrier and neurovascular unit by lipopolysaccharide-induced neuroinflammation. Int J Mol Sci. (2023) 24(2):1640. 10.3390/ijms24021640 36675154 PMC9862046

[32] Manouchehrian O Ramos M Bachiller S Lundgaard I Deierborg T. Acute systemic LPS-exposure impairs perivascular CSF distribution in mice. J Neuroinflammation. (2021) 18(1):34. 10.1186/s12974-021-02082-6 33514389 PMC7844902

[33] Bakhtadze MA Vernon H Karalkin AV Pasha SP Tomashevskiy IO Soave D. Cerebral perfusion in patients with chronic neck and upper back pain: preliminary observations. J Manipulative Physiol Ther. (2012) 35(2):76–85. 10.1016/j.jmpt.2011.12.006 22257946

[34] Taccone FS Su F Pierrakos C He X James S Dewitte O Cerebral microcirculation is impaired during sepsis: an experimental study. Crit Care. (2010) 14(4):R140. 10.1186/cc9205 20667108 PMC2945121

[35] Zhou Z Ma Y Xu T Wu S Yang GY Ding J Deeper cerebral hypoperfusion leads to spatial cognitive impairment in mice. Stroke Vasc Neurol. (2022) 7(6):527–33. 10.1136/svn-2022-001594 35817499 PMC9811541

[36] Xu F Xin Q Ren M Shi P Wang B. Inhibition of piezo1 prevents chronic cerebral hypoperfusion-induced cognitive impairment and blood brain barrier disruption. Neurochem Int. (2024) 175:105702. 10.1016/j.neuint.2024.105702 38401846

[37] Huang X Hussain B Chang J. Peripheral inflammation and blood-brain barrier disruption: effects and mechanisms. CNS Neurosci Ther. (2021) 27(1):36–47. 10.1111/cns.13569 33381913 PMC7804893

[38] Taccone FS Su F De Deyne C Abdellhai A Pierrakos C He X Sepsis is associated with altered cerebral microcirculation and tissue hypoxia in experimental peritonitis. Crit Care Med. (2014) 42(2):e114–122. 10.1097/CCM.0b013e3182a641b8 24196192

[39] Liu C Cardenas-Rivera A Teitelbaum S Birmingham A Alfadhel M Yaseen MA. Neuroinflammation increases oxygen extraction in a mouse model of Alzheimer’s disease. Alzheimers Res Ther. (2024) 16(1):78. 10.1186/s13195-024-01444-5 38600598 PMC11005245

[40] Gordon GR Choi HB Rungta RL Ellis-Davies GC MacVicar BA. Brain metabolism dictates the polarity of astrocyte control over arterioles. Nature. (2008) 456(7223):745–9. 10.1038/nature07525 18971930 PMC4097022

[41] Rancillac A Rossier J Guille M Tong XK Geoffroy H Amatore C Glutamatergic control of microvascular tone by distinct GABA neurons in the cerebellum. J Neurosci. (2006) 26(26):6997–7006. 10.1523/JNEUROSCI.5515-05.2006 16807329 PMC6673912

[42] Devor A Hillman EM Tian P Waeber C Teng IC Ruvinskaya L Stimulus-induced changes in blood flow and 2-deoxyglucose uptake dissociate in ipsilateral somatosensory cortex. J Neurosci. (2008) 28(53):14347–57. 10.1523/JNEUROSCI.4307-08.2008 19118167 PMC2655308

[43] Wang Y Tsytsarev V Liao LD. *In vivo* laser speckle contrast imaging of 4-aminopyridine- or pentylenetetrazole-induced seizures. APL Bioeng. (2023) 7(3):036119. 10.1063/5.0158791 37781728 PMC10541235

[44] Tsao CH Wu KY Su NC Edwards A Huang GJ. The influence of sex difference on behavior and adult hippocampal neurogenesis in C57BL/6 mice. Sci Rep. (2023) 13(1):17297. 10.1038/s41598-023-44360-8 37828065 PMC10570284

[45] Clayton JA. Studying both sexes: a guiding principle for biomedicine. FASEB J. (2016) 30(2):519–24. 10.1096/fj.15-279554 26514164 PMC4714546

